# Early Reputation Management: Three-Year-Old Children Are More Generous Following Exposure to Eyes

**DOI:** 10.3389/fpsyg.2018.00698

**Published:** 2018-05-15

**Authors:** Caroline Kelsey, Tobias Grossmann, Amrisha Vaish

**Affiliations:** Department of Psychology, University of Virginia, Charlottesville, VA, United States

**Keywords:** cooperation, orosocial behavior, reputation, self-presentation, watching eyes

## Abstract

To enhance their reputations, adults and even 5-year-old children behave more prosocially when being observed by others. However, it remains unknown whether children younger than five also manage their reputations. One established paradigm for assessing reputation management is the ‘watching eyes paradigm,’ in which adults have been found to be more prosocial in the presence of eyes versus control images. However, the robustness of this effect in adults has recently been called into question, and it has never been demonstrated in children. In Study 1, we used a method similar to that used in prior work: 3- and 5-year-old children took part in a prosocial task while in the presence of an image of eyes or flowers but without explicit mention or reference to the image. With this method, children did not show the watching eyes effect. In Study 2, 3-year-old children were tested with a modified watching eyes paradigm, wherein they first explicitly interacted either with images of eyes or with cloth flowers, and they then engaged in a prosocial task. With this modified watching eyes paradigm, 3-year-olds showed the predicted effect: They were more prosocial following exposure to eyes than flowers. These results offer potential insight into the mixed findings across the adult literature, such that the manner of exposure, and specifically how explicit the exposure is, may influence the watching eyes effect. Finally, no study to date has examined whether cues of human presence other than the eyes also influence prosociality. We found that children in the Mouth condition were prosocial at an intermediate level between the Eyes and Flowers conditions. Overall, the findings point to the remarkably early emergence of reputation management in human ontogeny.

## Introduction

Humans are an extremely cooperative species. Strikingly, we cooperate not only with family members but also with perfect strangers, and even when we cannot expect direct reciprocity (Nowak and Sigmund, 1998; Fehr and Fischbacher, 2003; Gintis et al., 2003). Such cooperation is thought to be maintained in part by the reputational costs that individuals incur when they break cooperative norms (Wedekind and Milinski, 2000; Milinski et al., 2002). As a result, adults engage in reputation management such that they act more prosocially when being watched by others (Goffman, 1959; Bull and Gibson-Robinson, 1981; Kurzban, 2001). However, much less is known about when children’s prosocial behavior is first motivated by reputational benefits.

Children engage in prosocial behaviors such as helping, comforting, and sharing from as early as the second year of life (e.g., Warneken and Tomasello, 2006; Svetlova et al., 2010). Recent research examining the motivations underlying such behaviors suggests that there are a wide variety of non-selfish reasons for children’s prosocial behaviors, ranging from concern for others’ plight to a desire to follow and enforce social and moral norms (Svetlova et al., 2010; Schmidt and Sommerville, 2011; Paulus, 2014; Eisenberg et al., 2016; Yucel and Vaish, 2018).

Importantly, later in development, children may also act prosocially for more self-oriented reasons, such as to gain or maintain a positive reputation with observers (Engelmann et al., 2012; Leimgruber et al., 2012). Early evidence for this came from interview studies, which showed that children begin to linguistically express their concerns about their reputations by 8 years of age, whereas 5-year-old children do not express such concerns (Aloise-Young, 1993; Banerjee et al., 2010). It was proposed that although 5-year-olds possess the cognitive ability required for reputation management (namely, second-order reasoning, or thinking about what others think of them), they are not yet concerned about their reputations and thus do not engage in reputation management (Banerjee and Yuill, 1999; Banerjee, 2002). However, more recently, researchers have argued that 5-year-olds may in fact have the motivation to manage their reputations but struggle with the self-awareness and linguistic skills required of them in previous work (Engelmann et al., 2012). Indeed, newer research shows that 5-year-old children help and donate more when being watched by others (Engelmann et al., 2012; Leimgruber et al., 2012). Current evidence thus suggests that reputation management in the form of increased prosocial behavior emerges around 5 years.

Surprisingly, no prior work has considered whether children younger than five also manage their reputations, though there is reason to think they might. For instance, 3-year-olds make reputational judgments about others (e.g., Olson and Spelke, 2008; Vaish et al., 2010) and they lie after cheating, perhaps to present themselves in a positive light (Evans and Lee, 2013). In addition, 3-year-olds experience guilt about violating moral norms, suggesting that they view and evaluate themselves according to social standards and through others’ eyes (Vaish and Tomasello, 2014; Vaish et al., 2016). Thus, although 3-year-olds may not yet possess the explicit and full-fledged second-order reasoning of older children (Sullivan et al., 1994; Talwar and Lee, 2008), they nonetheless seem capable, at least implicitly, of taking an observer’s perspective on themselves and judging their own actions according to external standards (Vaish and Tomasello, 2014). We may therefore expect that 3-year-olds also attempt to manage their reputations. The present studies were designed to test this prediction. Identifying when in ontogeny reputation management emerges can inform our understanding of when children begin to consider others’ perspectives on themselves and also further elucidate why young children engage in prosocial behavior.

To assess reputation management in young children, we relied on the ‘watching eyes’ effect, in which adults are more prosocial in the presence of images of eyes versus control images (e.g., flowers), arguably because the presence of eyes serves as an implicit signal of being watched and so triggers reputational concerns even in the absence of actual observers. For instance, Haley and Fessler (2005) assessed the donation behaviors of adults during a one-shot anonymous dictator game conducted on a desktop computer. During the task, participants were exposed either to stylized eyes or to a control, non-social image (the name of the lab) on the desktop background. Participants in the eyes condition donated significantly more money and were more likely to donate compared to those in the control condition. Similarly, Bateson et al. (2006) found that adults contributed significantly more money in the presence of eye images than flower images. This effect has been demonstrated across a wide range of real life and laboratory settings (Ernest-Jones et al., 2011; Powell et al., 2012). However, the robustness of the effect has recently been called into question by studies that have failed to find the effect (see Northover et al., 2017, for a review).

Moreover, to our knowledge, no prior work has successfully demonstrated the watching eyes effect in children. One study by Vogt et al. (2015) tested the effect among 5- and 8-year-olds using a one-shot dictator game. Children were presented with resources to distribute on the bottom half of a laminated pad, while the top half of the pad showed either a stylized drawing of eyes or a meaningless geometric pattern. Vogt et al. (2015) found no effect of the presence of eyes on mean donation amounts or likelihood of donation at either age. Similarly, in a study by Fujii et al. (2015), 5-year-old children were no more generous while playing a one-shot dictator game in the presence of stylized eyes versus flowers, though they were more generous when an experimenter was present and monitoring them. Based on their findings, Fujii et al. (2015) concluded that children’s increased generosity in the presence of others is not due to reputational concern (since the eyes did not lead to increased prosociality) but rather a concern about how a live observer would respond to their behavior. More generally, these two prior studies suggest that the presence of eyes is not sufficient to elicit reputation management behavior in children.

It is possible, however, that the lack of effects in these studies was due to methodological reasons. For instance, both prior studies utilized stylized images of eyes, which may be challenging for children to decipher. Prior research suggests that 3-year-old children are better able to understand and identify what an image is meant to represent when the image is more iconic (i.e., it more closely resembles the actual object, such as a photograph), as compared to a less iconic (more abstract) image (Callaghan, 2000). Children as young as 3 years also share more with a social partner who uses more naturalistic, communicative eye gaze cues (alternating gaze between children and an item of interest) compared to a partner who looks randomly around the room (Wu et al., 2018). Therefore, it is plausible that children would benefit from the use of realistic images of eyes as opposed to stylistic images of eyes.

It is also unclear whether children in the two previous studies noticed and paid attention to the images prior to making their prosocial decisions, or whether they were perhaps too focused on the prosocial task to do so. Indeed, Vogt et al. (2015) noted that several children in their study failed to follow the experimenter’s instructions because they were so engrossed in the distribution task, which raises the possibility that these children also did not pay much attention to the image of eyes or flowers. Fujii et al. (2015) did confirm that children saw the image before making their prosocial decision, but perhaps this brief and passive exposure did not impact children sufficiently. It is thus possible that in order for young children to show a watching eyes effect, the presentation of the eyes needs to be more explicit and interactive than in prior work, and needs to precede the donation task rather than be presented simultaneously so as to ensure that children can focus on one piece of information at a time (see Over and Carpenter, 2009).

The present work was designed to test these ideas. In Study 1, we tested children with a method resembling the watching eyes paradigm typically used with adults and used by Fujii et al. (2015) and Vogt et al. (2015), wherein 3- and 5-year-old children made a donation decision while in the presence of an image of eyes or flowers. However, we used photographs of eyes rather than stylized eyes in order to assess whether more realistic images of eyes elicit a watching eyes effect in children. In Study 2, we developed a more child-friendly watching eyes method wherein 3-year-old children were exposed to photographs of eyes or control stimuli in an interactive manner that ensured that they attended to the condition of interest. Moreover, it was only after this exposure that children made their donation decisions. This ensured that children first focused on the condition and then on the donation task.

For Study 1, our predictions were not very strong. On the one hand, the mixed watching eyes findings with adults and null findings thus far with children might lead us not to expect the watching eyes effect among 3- and 5-year-olds. On the other hand, given that 5-year-old children do increase their prosocial behavior in the presence of others (Engelmann et al., 2012; Leimgruber et al., 2012; Fujii et al., 2015), and that realistic images of eyes might be more effective in eliciting reputation management among children, we might expect that 5-year-olds will show greater generosity in the presence of images of eyes than flowers. Moreover, given that 3-year-old children show some evidence of presenting themselves in a positive light and viewing their own actions through the eyes of others (Evans and Lee, 2013; Vaish and Tomasello, 2014; Vaish et al., 2016), we might expect that even 3-year-olds will also show a watching eyes effect.

For Study 2, we predicted that our modifications to the watching eyes paradigm would increase children’s likelihood of attending and responding to the condition. We thus predicted that 3-year-old children, who, as argued above, do at least implicitly have the capacity to evaluate themselves and present themselves in a positive light, would be more generous if they had been explicitly and interactively exposed to eyes compared with flowers. Note that as a recent meta-analysis of the watching eyes effect revealed that the presence of eyes primarily influences the initiation of donation behavior rather than the amount of resources donated (Nettle et al., 2013), we expected that the effect of eyes might primarily emerge in the likelihood of children sharing rather than the amount they share.

Interestingly, no study to date, whether with adults or with children, has examined whether other cues of human presence also motivate prosocial behavior or whether the eyes are special in this regard because they most clearly signal being observed. In one study relevant to this question, Haley and Fessler (2005) tested whether adults who wore noise-reducing headphones (and thus received fewer auditory cues of human presence) were less generous in a dictator game. They did not find an effect of headphones on donation behavior, suggesting that eyes are perhaps especially effective at eliciting reputational concerns. Another study found that adults exposed to eyes (photos portraying only the eye regions of statues) donated significantly more money than individuals in a more general social condition (pictures of people facing away from the participant) and individuals in a non-social control condition (pictures of empty school hallways; Baillon et al., 2013). Regarding the watching eyes effect in particular, a series of experiments examined whether adults exposed to “watching” eyes (i.e., eyes with direct gaze) were more likely to help others, compared to individuals exposed to closed or averted eyes. The findings from the individual experiments were inconsistent. However, when the results from the three experiments were pooled, there was some evidence that participants were more prosocial in the presence of watching eyes than the other eye stimuli (Manesi et al., 2016). Given these tentative conclusions and as no other work thus far has included other human features, more research with closer-matched comparisons of other signs of human presence is warranted. Furthermore, others have suggested that flowers are not an adequate control as exposure to flowers may increase the participants’ positivity and this in turn may impact their prosocial behavior (Raihani and Bshary, 2012). Thus, in Study 2, we also tested the effect of a second human facial feature – the mouth, which, like the eyes, is a human facial feature that serves social functions, yet it does not signal being watched as clearly as the eyes (and thus may not trigger reputational concerns to the same degree as eyes). However, considering the novelty of using mouths in this experimental context, we did not have a specific prediction about the effect of the mouth exposure on children’s prosocial behavior.

## Study 1

### Method

#### Participants

A total of 64 children were included in the study, of which half were 5-year-olds (*n* = 32; 16 females; age range: 60–71 months; *M_age_* = 64.56 months, *SD* = 3.25 months) and half were 3-year-olds (*n* = 32; 16 females; age range: 36–47 months; *M_age_* = 41.01 months, *SD* = 3.28 months). Participants were recruited from a mid-Atlantic university town. The majority of participants were Caucasian (83.1% Caucasian, 5.1% Black, 5.1% Asian, and 6.8% Other) and had parents with high levels of education (50.0% post graduate, 35.4% college graduate, and 14.5% high school graduate). Three additional participants were tested but excluded because they were distracted and could not follow the procedure (*n* = 2) or did not want to take part in the distribution task (*n* = 1).

#### Materials and Procedure

This study was conducted as part of a larger study on children’s responses to social cues. Children were randomly assigned to one of two conditions (16 children of each age group in Eyes and Flowers). The experimenter placed a small box in front of the child and said, “Oh look, a box, I wonder what’s inside. Do you want to open the box?” Inside the lid of each box was a 6 × 12 cm photograph of either eyes or flowers, and in the box were four colored, crayon-shaped erasers (see Supplemental Materials for more information and example figures of each crayon box). The experimenter held the lid of the box open so that the picture was facing the child during the sharing task instructions. The experimenter explained that these four erasers were for the child for playing with her, but the child could choose to share these erasers with the next child who was going to play the game. The experimenter gave the child a white envelope and told her this was the child’s envelope, and that the child could put “any of the erasers you want to keep into your white envelope.” The experimenter then showed the child a red envelope and told her this was for the next child, and the child could put “any of the erasers you want to share into this red envelope.” Finally, it was emphasized to the child to “decide all by yourself” which erasers to keep and which to share, and that the experimenter and the parent would not know of their decision. Children had to pass comprehension questions about how many erasers there were and which envelope was for which child. If the child answered any of the comprehension questions incorrectly, instructions were repeated and the child was asked again. Throughout, the experimenter did not make any reference to the photograph of eyes or flowers inside the eraser box. If the child spontaneously mentioned the photograph, the experimenter did not acknowledge the content of the photo and instead continued with the explanation of the eraser distribution task.

The experimenter then left the room and returned once the child had made the decision (the experimenter watched without being noticed from outside the room). During this time, the lid of the crayon box was left open so that the picture of the eyes or flowers was visible during the distribution task. Note that for this study, parents were invited to stay in the room for the duration of the procedure. This decision could potentially have elicited unintended reputational concerns among the participants. However, to minimize this possibility, parents were always seated out of view of the child and kept busy by completing questionnaires throughout the procedure. Additionally, given that parents were present during both Eyes and Flowers conditions, any condition difference that emerges cannot be attributed to parental presence as it was identical in both conditions.

Three different experimenters (two of which were blind to hypotheses) carried out the testing across the sample. Analyses conducted after the experiment revealed no differences in results across experimenters (*p* = 0.91). All children received a small toy for their participation. This study was carried out in accordance with the recommendations of the authors’ institutional IRB. This study was approved by the authors’ institutional IRB. All parents gave written informed consent in accordance with the Declaration of Helsinki. The protocol was approved by the authors’ institutional IRB.

### Results

#### Likelihood of Sharing

**FIGURE 1:**
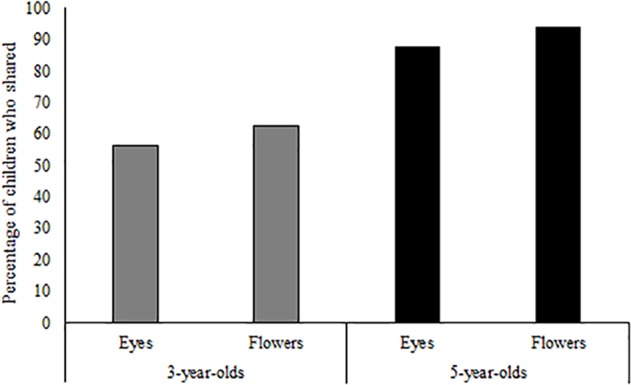
The percentages of 3- and 5-year-olds in the original (implicit) “watching eyes” paradigm who shared.

To assess whether children’s sharing differed across conditions, data were first dichotomized to reflect sharing or no sharing. A binary logistic regression was conducted with age group (3-year-olds vs. 5-year-olds), condition (Flowers vs. Eyes), and the age by condition interaction predicting likelihood of donation (0 vs. 1–4 erasers). However, neither the main effects of age group (*p* = 0.58) or condition (*p* = 0.90), nor the interaction between age group and condition (*p* = 0.73) predicted the likelihood of sharing (see **Figure 1**). Specifically, 14 5-year-olds (87.5%) and 9 3-year-olds (56.3%) shared at least one eraser in the Eyes condition, and 15 5-year-olds (93.8%) and 10 3-year-olds (62.5%) shared at least one eraser in the Flowers condition.

#### Number of Erasers Shared

To further assess differences across conditions, a 2 × 2 ANOVA was conducted to test if age group, condition, or the age group by condition interaction predicted the total number of erasers shared. This revealed a trend for age group, such that 5-year-olds (*M* = 1.66, *SD* = 0.79) shared somewhat more erasers than 3-year-olds (*M* = 1.22, *SD* = 1.13), *F*(1,60) = 3.22, *p* = 0.078, $\eta_{p}^{2}$ = 0.05. However, there was no main effect of condition (*p* = 0.45) and no interaction between age group and condition (*p* = 0.31). Specifically, in the Eyes condition, 5-year-olds shared an average of 1.69 erasers (*SD* = 0.95) and 3-year-olds shared an average of 1.00 erasers (*SD* = 0.97), and in the Flowers condition, 5-year-olds shared an average of 1.63 erasers (*SD* = 0.62) and 3-year-olds shared an average of 1.44 erasers (*SD* = 0.1.26). Note that a negative binomial regression with a link function was also calculated in order to account for the count structure of the sticker distribution. This yielded similar results: no main effects of age or condition and no age by condition interaction (Akaike’s Information Criterion [AIC] = 217.75; all *p*-values > 0.33).

### Discussion

In Study 1, we found that 5-year-olds showed a tendency to share more erasers than 3-year-olds and that on average, 5-year-olds shared close to half of their erasers (1.67 of 4). This pattern is in line with prior work on sharing behavior in young children (e.g., Rochat et al., 2009). However, regarding our central question about children’s reputation management behavior, we found that neither 3- nor 5-year-old children acted more prosocially in the presence of a photograph of eyes versus flowers.

The lack of difference in sharing across the Eyes and Flowers conditions is consistent with the two prior studies that tested for a watching eyes effect in children that also did not find evidence of the effect (Fujii et al., 2015; Vogt et al., 2015). Although this lack of effect could be taken as evidence that preschool-aged children do not manage their reputations in the presence of minimal monitoring cues such as images of eyes, it is also possible that children in Study 1 simply did not attend to the images because they were too engrossed in the prosocial task, which resulted in the images not having an effect on their prosocial behavior. We thus reasoned that young children might show the predicted watching eyes effect if the exposure were more explicit and interactive than in prior work, and if the exposure preceded the prosocial task so as to ensure that children can focus on one piece of information at a time. Moreover, in Study 1, only one exemplar of eyes was used (a Caucasian female with blue eyes), raising the possibility that the null effects are limited to this particular exemplar. Therefore, in Study 2, we included both female and male eyes, along with different colors of eyes.

Ultimately, we were interested in examining when children are first sensitive to reputational cues. Study 2 was designed to test this question, and was conducted just with 3-year-old children based on our prediction that children of this age possess the fundamental capacities required to manage their reputations. Furthermore, we chose not to include 5-year-olds in Study 2 for an important psychometric reason. Specifically, in Study 1, we saw a lack of variability in the likelihood of sharing among 5-year-olds (nearly 91% of 5-year-olds shared at least one eraser across conditions). Given that a recent meta-analysis of the watching eyes effect found that the presence of eyes primarily influences the initiation of donation behavior rather than the amount of resources donated (Nettle et al., 2013), we reasoned that the near-ceiling likelihood of sharing among 5-year-olds might preclude the detection of an effect in this age group. On the other hand, 3-year-olds in Study 1 showed a far more variable likelihood of sharing (around 60% of 3-year-olds shared at least one eraser across conditions). We thus focused on 3-year-olds in Study 2.

Furthermore, it could be argued that Study 1 lacked adequate power. Although Study 1 had a similar sample size to other studies that have used similar exposure procedures (e.g., Over and Carpenter, 2009; Piazza et al., 2011), it is possible, given the small effects of implicit monitoring cues reported in previous research, that more participants would be needed to observe a potentially small effect (Fujii et al., 2015). Thus, for Study 2 we made an a-priori decision to set the sample size at 84 children; this sample size was selected based on sample sizes used by similar studies of reputation management (e.g., Engelmann et al., 2012) and confirmed by a power analysis conducted by G^∗^power based on the detection of a medium effect size. Finally, as no prior work has examined the effect of other human features on prosocial behavior and there is a need to include closer matched controls, Study 2 also included a third condition in which children were exposed to images of the mouth.

## Study 2

### Method

#### Participants

A total of 84 children were included in the study (42 males and 42 females; age range 36–47 months, *M_age_* = 41.42 months, *SD* = 3.32 months). The participants had similar racial diversity (84.8% Caucasian, 1.3% Black, 1.3% Asian, and 12.7% Other) and parental education levels (55.0% post graduate, 38.9% college graduate, and 6.3% high school graduate) to participants in Study 1. Nineteen additional participants were tested and excluded because they were distracted and could not follow the procedure (*n* = 14), they did not distribute the stickers (*n* = 4), or the parent interfered with the study (*n* = 1).

#### Materials and Procedure

Children were randomly assigned to one of three conditions (28 each in Eyes, Mouth, and Flowers). During the exposure phase, children first had a “lesson” about their assigned condition during which the experimenter asked them five questions about the item, such as about its function (e.g., “What do you do with your eyes?”) and location (e.g., “Where are your eyes?”). Note that for the Eyes and Mouth conditions, the exposure phase referenced the children’s own eyes or mouth, and for the Flowers condition, it referenced cloth flowers that the children held. After the short lesson, there was a matching game involving three pairs of pictures of eyes, mouths, or flowers (depending on condition). Children were asked to find the matching pairs and the experimenter described characteristics of the matched pairs (e.g., colors and number). After the matching game, the pictures were left facing up and were visible to the child during the sharing task. The details of the lesson and matching game are provided in the Supplementary Materials.

After this exposure phase, children began the sticker distribution task. The experimenter placed four stickers in front of the child and said that these were for the child for playing the game. The directions and comprehension tasks were the same as for the eraser-sharing task in Study 1. The only differences between this task and the sharing task of Study 1 were in the items being shared (crayon-shaped erasers in Study 1, stickers in Study 2) and the fact that there was no box with a picture of the condition item in Study 2. All children received a small toy for their participation. Three different experimenters (two of which were blind to hypotheses) carried out the testing across the sample. Analyses conducted after the experiment revealed no differences in results across experimenters (*p* = 0.44). This study was carried out in accordance with the recommendations of the authors’ institutional IRB. This study was approved by the authors’ institutional IRB. All parents gave written informed consent in accordance with the Declaration of Helsinki.

### Results

#### Likelihood of Sharing Stickers

**FIGURE 2:**
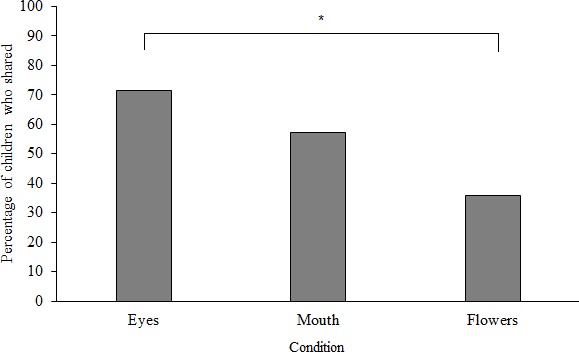
The percentages of 3-year-olds in the explicit “watching eyes” paradigm who shared. Note: ^∗^*p* < 0.05.

To assess whether children’s sharing differed across conditions, data were first dichotomized to reflect sharing or no sharing. In support of our hypothesis, the likelihood of sharing was significantly different across conditions, *X^2^*(2, *N* = 84) = 7.30, *p* = 0.026, *V* = 0.30. Specifically, 20 (71.4%) children shared in the Eyes condition, 16 (57.1%) shared in the Mouth condition, and 10 (35.7%) shared in the Flowers condition. Follow-up tests revealed a significant difference in sharing only between the Eyes and Flowers conditions, *X^2^*(1, *N* = 56) = 7.18, *p* = 0.007, φ = 0.36. The Mouth condition did not differ significantly from the other two conditions (see **Figure 2**).

#### Number of Stickers Shared

To further assess differences across conditions, we compared the number of stickers shared. The mean number of stickers shared in each condition followed the predicted pattern, with the greatest number shared in the Eyes condition (*M* = 1.46, *SD* = 1.23), an intermediate number in Mouth (*M* = 1.18, *SD* = 1.28), and the fewest in Flowers (*M* = 0.86, *SD* = 1.30). However, a comparison across all three conditions was not significant, Kruskal–Wallis Test, *p* = 0.12. A negative binomial regression with a link function was also performed, which confirmed this result. Specifically, there were no significant differences in number of stickers shared between the Eyes and Mouth conditions (AIC = 181.34; *p* = 0.54), or between the Eyes and Flowers conditions (AIC = 168.97; *p* = 0.15).

### Discussion

Study 2 found that children as young as 3 years of age engage in reputation management. More specifically, 3-year-olds were more likely to show prosocial behavior when they had been exposed in an explicit and interactive manner to photographs of eyes as opposed to flowers. Furthermore, the results raise interesting questions about the impact of general human presence, as children who were exposed to photographs of mouths shared to an intermediate degree between the Eyes and Flowers conditions but their sharing did not differ significantly from either condition. It is possible, however, that a substantially larger sample size than that of Study 2 would be needed to detect a small but significant difference between the Mouth condition and one of the other two conditions; this will be important to address in future research.

These results provide the first evidence of a watching eyes effect in young children, and the first rudimentary evidence of reputation management in children younger than 5 years of age. Moreover, they tentatively suggest that the eyes are an especially effective human facial feature for eliciting prosocial behavior. Thus, reputation management in the context of watching eyes seems to already be present in early childhood.

## General Discussion

Managing one’s reputation is a critical mechanism underlying human cooperation (Wedekind and Milinski, 2000; Milinski et al., 2002). Prior studies have found that children as young as 5 years of age manage their reputations by exhibiting greater prosocial behavior when being observed by others (Engelmann et al., 2012; Leimgruber et al., 2012). However, no studies to date have examined whether reputation management occurs even earlier in development. We hypothesized that reputation management may be evident as early as 3 years of age given that children of this age make reputational judgments about others, attempt to present themselves in a positive light, and seem capable of seeing and evaluating themselves from the perspective of others (Olson and Spelke, 2008; Evans and Lee, 2013; Vaish and Tomasello, 2014; Vaish et al., 2016).

In addition, no prior work has demonstrated the watching eyes effect in children, i.e., demonstrated that children manage their reputations even in the presence of minimal cues of monitoring such as images of eyes. To our knowledge, only two prior studies had set out to examine this effect in children but found no evidence that 5- and 8-year-old children’s prosocial behavior increased in the presence of eyes versus a control image (Fujii et al., 2015; Vogt et al., 2015). As these prior studies had used stylized eyes rather than photographs of eyes, we reasoned that children might not have interpreted the images as eyes and thus not been motivated to manage their reputations in response. However, in our Study 1, when 3- and 5-year-old children made a sharing decision in the presence of a photograph of eyes versus flowers, we still did not find a difference in sharing behavior. Consequently, we asked whether the lack of a watching eyes effect among children in Study 1 and in prior work might be because children were so focused on the prosocial task that they did not pay much attention to the image of eyes, and were thus not motivated to engage in greater prosocial behavior in order to manage their reputations.

For Study 2, we devised a more child-friendly version of the watching eyes paradigm wherein 3-year-olds were first exposed in an explicit and interactive way with eyes or flowers, and then made a sharing decision toward an unknown peer. As expected, children were significantly more likely to share stickers if they had been exposed to eyes than flowers. The watching eyes effect is premised on the idea that the eyes signal that someone is being monitored and therefore, increased prosocial behavior in response to eye cues can be attributed to eyes serving as cues to engage in reputation management (Haley and Fessler, 2005). A similar interpretation, in terms of reputation management, has been offered in previous research in which 5-year-old children showed increases in prosocial behavior in the context of peer presence or peer visibility (Engelmann et al., 2012; Leimgruber et al., 2012). In line with these prior interpretations of research with adults and children, we argue that the current results can be taken as initial evidence that children show evidence of reputation management behavior by as early as 3 years of age.

Study 2 also provides the first demonstration of the watching eyes effect in children. We think it is possible that previous failures to find the effect with children might be at least partially explainable by the methodology used in the typical watching eyes paradigm (versions of which were used in the previous studies with children, including our Study 1). More specifically, the typical watching eyes paradigm and the one used in Study 1 passively present an image of eyes during a prosocial task, which may not draw children’s attention or engage them sufficiently to elicit reputation management behaviors. In Study 2, these problems were attenuated by the modifications that we made to the typical paradigm, including presenting the images explicitly and having children interact with them, and doing so before they made their prosocial decisions. These modifications were effective, thus providing evidence that even young preschoolers increase their prosocial behavior when exposed to cues of being monitored.

It should be noted that one of the reasons that the watching eyes effect is so striking and has received so much attention in the field is that in the typical watching eyes paradigm, the presentation of the eyes is subtle and tacit (e.g., on the background of the desktop on which participants are making prosocial decisions; Haley and Fessler, 2005). The fact that such subtle cues can elicit reputation enhancement behavior has been taken as evidence that humans are evolutionarily adapted to attend to and be influenced by implicit cues of observability, even outside of explicit reasoning about reputation (Haley and Fessler, 2005). As such, our modified procedure in Study 2, in which the eyes (or flowers) were explicitly discussed in a ‘lesson’ and children played a game with the images, might be seen to be moving away from this strong argument about the intuitive and implicit influences on reputation management. However, note that our modified procedure with the eyes did not involve any mention of the child being monitored or of reputation formation, and although the ‘lesson’ about the eyes did involve reference to the functions of eyes (“seeing”), no reference was ever made to the child being seen or watched. Thus, children’s increased prosociality after being exposed to eyes cannot be explained by children having explicitly reasoned about reputation. Importantly, even if children did reason explicitly about reputation as a result of our modified procedure, the results are still impressive in suggesting that children as young as 3 years may engage in reputation management.

The procedure of Study 2 involved another notable modification to the watching eyes effect, namely, having participants think about their own eyes (such as about what they do with their eyes). Thinking about one’s own eyes (or any other feature of the self) may increase positive affect or attention to the task (Hardy and Oliver, 2014), and it could be argued that this is what led to the observed increase in children’s prosocial behavior. However, this is unlikely to fully explain our results, for several reasons. First, interacting with flowers has also been shown to increase positive affect and mood (Haviland-Jones et al., 2005). Second, in the Mouth condition, children also thought about their own mouths, but this did not significantly increase their prosocial behavior compared to the Flowers condition. Third, note that the matching game, which immediately preceded the prosocial task, involved children thinking about and interacting with images of others’ eyes rather than their own. Finally, the photographs of others’ eyes were present throughout the prosocial task. For these reasons, we think it is unlikely that children’s increased prosocial behavior resulted only from thinking about their own eyes. However, a more systematic examination of this possibility will be interesting to pursue in future work.

Our finding that 3-year-old children behaved more prosocially in response to eye cues is in line with previous research showing that children from a young age are able to detect and respond to social information from eye cues (Farroni et al., 2002; Grossmann, 2017; Wu et al., 2018). This privileged attention and sensitivity to eyes is argued to allow infants and young children to detect the presence and some of the contents of other minds (Grossmann, 2017). Moreover, a preferential sensitivity to eyes begins in infancy and continues into adulthood, and at least among adults, attentiveness to eye cues is predictive of an individual’s prosocial behavior in a reputation-relevant setting (Vaish et al., 2017). An early-emerging sensitivity to eyes may thus be a vital step toward understanding others’ minds and seeing the self through the filter of those minds, which in turn are both necessary components of managing one’s reputation.

An open question is whether even children younger than 3 years might manage their reputations. To answer this, it is important to consider how reputation management behavior may develop. Prior work suggests that 2-year-olds do not yet engage in behaviors such as lying after they have cheated (Evans and Lee, 2013). They also do not show signs of guilt after transgressing, and thus seem not to view and evaluate themselves through others’ eyes (Vaish et al., 2016). Based on these prior findings, we may predict that reputation management first emerges only around 3 years of age. On the other hand, by around 1.5 to 2 years of age, children pass the mirror self-recognition task and display the self-conscious emotion of embarrassment when they notice that others can see them in the mirror, which is thought to reflect the developmental emergence of self-awareness, requiring at least a basic sense of how they appear to others (Lewis et al., 1989; Lewis and Ramsay, 2004). It is thus possible that reputational concern does emerge even earlier than seen in the current study. Even if reputational concern does emerge along with the earliest instances of self-awareness, however, it is unclear whether this concern leads such young children to care about and actively *influence* others’ impressions of them (i.e., engage in reputation management) or whether that motivation emerges only later in ontogeny.

It is important to mention here that we did attempt to pilot our Study 2 procedure with a few 2-year-old children. However, these young children seemed not to grasp the basics of the sticker sharing task. For instance, they often did not respond to the questions that the experimenter posed as part of the “lesson” and could not reliably identify which envelope was for them versus for the next child. Future work could, however, adapt and simplify our task for children younger than 3 years in order to shed light on the developmental emergence of reputation management.

Importantly, in addition to investigating whether young children manage their reputations in the presence of eyes versus control, inanimate objects (flowers), Study 2 also asked whether eyes are unique among the human features to promote cooperative behaviors. Specifically, do eyes increase prosocial behavior because they signal that one is being watched and thus elicit reputational concerns, or simply because they signal the presence of others, which perhaps makes individuals feel more social and thus behave more cooperatively? If the latter is true, then cues of human presence other than the eyes may similarly enhance cooperative behavior. We thus also tested the effect of a mouth, as the mouth is a human facial feature that serves social functions, yet it does not signal being watched as clearly as the eyes.

Interestingly, the mouth elicited an intermediate level of prosocial behavior that was not significantly different from the eyes or the flowers. This hints at the possibility that while more general cues of human presence may edge prosocial behavior a little higher, it is the ‘watching’ function of the eyes – and thus the reputational concerns they elicit – that truly promotes prosocial behavior in a substantial way. This idea is supported by recent work with adults showing that eyes that “pay attention” (i.e., eyes with direct gaze) increase prosocial behavior more than eyes with averted gaze or closed eyes (Manesi et al., 2016). Therefore, it is not simply any indicator of human presence but specifically being watched that is associated with enhanced prosociality. Alternatively, it is possible that eyes are a more salient facial feature, and thus are more easily recognized as a human feature compared to a mouth (Keil, 2009). In other words, eyes may be a necessary feature to detect a face, whereas other features such as mouths may not be potent enough to index the presence of a face, and as a result, may not elicit reputational concerns. However, much more work is needed to clarify whether cues of human presence might enhance prosocial behavior, and if so, what mechanisms underlie the effect of the eyes versus the effect of other human features.

## Conclusion

We have shown here for the first time that even children as young as 3 years of age engage in reputation management. Of course, the ability to manage one’s reputation is likely rudimentary at this young age and becomes more sophisticated and flexible over development (Engelmann et al., 2012). Nonetheless, our findings demonstrate that one of the key mechanisms believed to underlie large-scale human cooperation is functional from remarkably early in ontogeny.

## Author Contributions

CK, TG, and AV designed the research, analyzed the data, and wrote the paper. CK performed research.

## Conflict of Interest Statement

The authors declare that the research was conducted in the absence of any commercial or financial relationships that could be construed as a potential conflict of interest.
