# Supplementary material for: Early Reputation Management: Three-Year-Old Children Are More Generous Following Exposure to Eyes
Source: Front Psychol. 2018 May 15;9:698. doi: 10.3389/fpsyg.2018.00698 (PMC5962684; doi:10.3389/fpsyg.2018.00698)
Supplement: Supplementary file 1 [file Data_Sheet_1.docx]

Early reputation management:

Three-year-old children are more generous following exposure to eyes

Supplemental Material


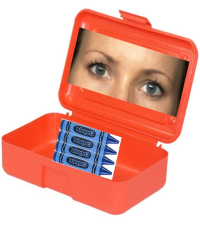

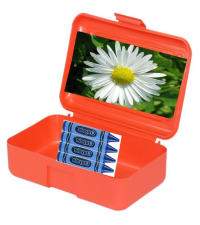
**Supplemental Figure**

A

B

*Supplemental Figure 1.* Example crayon boxes used for the Flowers (panel A) and Eyes (panel B) conditions.

**Method for exposure for Study 1**

**Materials for priming for Study 1.**

The picture of eyes was taken from a previously validated FACES database (http://faces.mpib-berlin.mpg.de). The area of the eyes was individually cropped from a photograph of a Caucasian female model displaying a neutral expression. The photograph of the daisy flower was taken from the Nilsback and Zisserman database (<http://www.robots.ox.ac.uk/~vgg/data/flowers/17/>).

**Method for exposure for Study 2**

**Script for “lesson” with images (of eyes, mouths, or flowers) for Study 2.**

Experimenter asked each question, waited 5 s for the child to answer, and then provided a reiteration after the child answered. For the Flowers condition, the child was given two white flowers to hold during the lesson.

Eyes:

Question 1: “What are these?” [pointing to experimenter’s eyes]

Reiteration: “Yes, those are the eyes!”

Question 2: “What do you do with your eyes?”

Reiteration: Experimenter ensured that ‘seeing’ was mentioned, as follows:

If child responded “see” or something similar: “Yes, you do see with your eyes!”

If child responded in some other way (e.g., “blink”): “Yes, you do [child’s response] with your eyes! And you see with your eyes!”

Question 3: “What do you see with your eyes right now?”

Reiteration: “Yes, you do see [child’s response] with your eyes right now!”

Question 4: “How many eyes do you have?”

Reiteration: Experimenter ensured that two eyes were mentioned, as follows:

If correct, “Yes, you do have two eyes!”

If incorrect, experimenter counted while pointing to eyes, “You have one, two eyes!”

Question 5: “Where are your eyes?”

Reiteration: “Yes, that is where your eyes are!”

Mouth:

Question 1: “What is this?” [pointing to experimenter’s mouth]

Reiteration: “Yes, that is the mouth!”

Question 2: “What do you do with your mouth?”

Reiteration: Experimenter ensured that ‘tasting’ was mentioned, as follows:

If child responded “taste” or something similar: “Yes, you do taste with your mouth!”

If child responded in some other way (e.g., “smile”): “Yes, you do [child’s response] with your mouth! And you taste with your mouth!”

Question 3: “What do you taste with your mouth right now?”

Reiteration: “Yes, you do taste [child’s response] with your mouth right now!”

Question 4: “How many mouths do you have?”

Reiteration: Experimenter ensured that one mouth was mentioned, as follows:

If correct, “Yes, you do have one mouth!”

If incorrect, experimenter counted while pointing to mouth, “You have one mouth!”

Question 5: “Where is your mouth?”

Reiteration: “Yes, that is where your mouth is”

Flowers:

Question 1: “What are these?” [pointing to two flowers that child was given to hold]

Reiteration: “Yes, those are flowers!”

Question 2: “What do you do with flowers?”

Reiteration: “Yes, you do [child’s response] with flowers!”

Question 3: “Where do flowers grow?”

Reiteration: “Yes, flowers do grow in [child’s response]!”

Question 4: “How many flowers do you have?”

Reiteration: Experimenter ensured that two flowers were mentioned, as follows:

If correct, “Yes, you do have two flowers!”

If incorrect, experimenter counted while pointing to flowers child was holding, “You have one, two flowers!”

Question 5: “Where are the flowers?”

Reiteration: “Yes, that is where the flowers are!”

**Matching game**

After the lesson, a matching game was played using images corresponding to the condition. Six cards (consisting of three matching pairs of eyes, mouths, or flowers, depending on condition) were placed face down on the table. The children were instructed to flip over two at a time to find the matching pairs. The experimenter commented on specific physical features (e.g., eye color or number of flower petals) to assist the child in finding the pair.

Pictures of facial features were taken from a previously validated FACES database (http://faces.mpib-berlin.mpg.de). The areas of the eyes and mouth were individually cropped from photographs of Caucasian male and female models displaying neutral expressions. The photographs of flowers were of different daisies and were taken from the Nilsback and Zisserman database (<http://www.robots.ox.ac.uk/~vgg/data/flowers/17/>).
